# Preferences of University Students for a Psychological Intervention Designed to Improve Sleep: Focus Group Study

**DOI:** 10.2196/44145

**Published:** 2023-08-24

**Authors:** Michelle Tadros, Sophie Li, Emily Upton, Jill Newby, Aliza Werner-Seidler

**Affiliations:** 1 The Black Dog Institute The University of New South Wales Randwick Australia; 2 School of Psychology The University of New South Wales Sydney Australia

**Keywords:** university students, sleep difficulties, intervention, student needs, insomnia, treatment, focus group, intervention design, sleep, sleep medicine, student, university, college, post secondary, psychological, psychotherapy, help-seeking, polysomnography

## Abstract

**Background:**

Many university students have difficulties with sleep; therefore, effective psychological treatments are needed. Most research on psychological treatments to improve sleep has been conducted with middle-aged and older adults, which means it is unclear whether existing psychological treatments are helpful for young adult university students.

**Objective:**

This study aimed to discover university student preferences for a psychological intervention to improve sleep quality.

**Methods:**

Focus groups were conducted over 3 stages to examine students’ views regarding content, format, and session duration for a psychological intervention to improve sleep. A thematic analysis was conducted to analyze participant responses.

**Results:**

In total, 30 participants attended small focus group discussions. Three key themes were identified: (1) program development, (2) help-seeking, and (3) student sleep characteristics. Program development subthemes were program format, program content, and engagement facilitators. Help-seeking subthemes were when to seek help, where to access help, stigma, and barriers. Student sleep characteristics subthemes were factors disturbing sleep and consequences of poor sleep.

**Conclusions:**

Students emphasized the need for a sleep intervention with an in-person and social component, individualized content, and ways to monitor their progress. Participants did not think there was a stigma associated with seeking help for sleep problems. Students identified the lack of routine in their lifestyle, academic workload, and the pressure of multiple demands as key contributors to sleep difficulties.

## Introduction

Sleep difficulties are common among university students, with 66% reporting some level of sleep disturbance [1]. Sleep disturbances typically include difficulty initiating sleep, frequent awakenings after sleep onset, early morning awakening, unrefreshing sleep, and short sleep duration [2]. At the more severe end of the sleep disturbance spectrum is insomnia disorder, which is defined by the Diagnostic and Statistical Manual of Mental Disorders Fifth Edition (DSM-V) [3] as dissatisfaction with the quantity or quality of sleep despite adequate opportunity for sleep, that occurs at least 3 times per week over the duration of at least 3 months [3]. Insomnia disorder has been found in 18.5% of university students [4], a prevalence rate much higher than the 6% [5] found in general adult populations. Sleep disturbance is associated with negative consequences such as poor academic performance [6-8] and impaired social functioning [9].

Sleep disturbance is also associated with the development of mental illness and suicidality [10,11]. Indeed, in recent decades, there has been an increasing awareness of the fundamental role sleep plays in maintaining mental health [12]. Evidence from the general population suggests that poor sleep predisposes individuals to mental illnesses such as depression and anxiety [12-15]. It can also heighten the risk of suicidal thoughts and behaviors [16], worsen comorbid mental health conditions, increase the likelihood of relapse [17], prolong the course of depression [18,19], and blunt treatment effects [20]. This makes the effective treatment of sleep disturbance a priority [21,22]. This is particularly important for university students who, being young adults typically aged 18-25 years, have an increased vulnerability to the onset of mental disorders [23]. It is well documented that this age group has a high prevalence of mental health disorders relative to other age groups [24]. Furthermore, it has been suggested that university students have higher rates of mental health disorders when compared to age-matched nonstudents [25].

Psychological interventions designed to improve sleep are effective in general adult populations [26-29], comparable to the pharmacological approaches typically used in primary care [30]. Cognitive behavioral therapy for insomnia (CBT-I) is the first-line psychological treatment recommended for insomnia by national and international peak bodies including The Australasian Sleep Association [31] and the American Academy of Sleep Medicine [32]. CBT-I is a multicomponent treatment package that is typically comprised of sleep hygiene education, sleep restriction, tension reduction techniques such as relaxation, cognitive therapy to target unhelpful beliefs about sleep, and techniques to help people deal effectively with their worries. A systematic review of 87 randomized controlled trials (RCTs) showed that CBT-I significantly improved sleep quality with large effect sizes (g=0.98) in adults (aged from 17 to 75 years) [33]. While CBT-I is well established as effective at improving sleep in the general population of adults [34], there is much less research into the effects of these interventions in university student groups [35,36].

To date, there have been 5 RCTs of CBT-I with university students [14,37-40]. Four of these studies used interventions designed for general adult populations, which do not consider the unique circumstances of university students [14,38-40]. University students’ living circumstances (in student dorms, share houses, or with their family of origin) often mean that they spend large amounts of time in their bedroom [41], this is contrary to typical sleep hygiene recommendations. Other factors disturbing their sleep include stress [1,42], irregular sleep-wake patterns [43], the lack of routine in their study timetables, and the tendency to stay up late to complete assignments or engage in social activities [41]. These aspects of university student life underscore the need for sleep interventions that specifically address their needs. Only 1 study used an intervention designed specifically for university students [37]. However, it was focused on students who had both sleep difficulties and excessive alcohol consumption. In this study, the intervention had a dual focus on both alcohol and sleep, limiting the relevance of the findings for more general student populations with sleep difficulties.

Other limitations of the available research include the use of small sample sizes [37,40], brief interventions designed for nonclinical populations [38], and low adherence to treatment [14]. Although speculative, it is possible that the use of generic programs that are not tailored to the needs of university students could have contributed to low adherence rates. The efficacy of CBT-I in this population cannot be firmly established without further research that overcomes these limitations.

User-centered design principles are being increasingly adopted in the development of mental health interventions [44]. This approach has roots in the fields of human-computer interaction, cognitive psychology, and industrial design. User-centered design principles espouse that effective programs are not simply adhering to evidence-based therapeutic components determined by expert clinicians but are also practical, convenient, memorable, efficient, and acceptable for those taking part in the intervention [45]. A user-designed development process involves consultation during the design process with the people who will be using the intervention, and harnessing their involvement to shape the features of the program being developed, including aspects such as format, content, timing, and delivery. Gaining an understanding of end user needs and preferences through a user-centered design approach is an important first step to developing a program that will be effective, practical, and accessible [45].

The aim of this study was to engage with university students to inform the development of a new sleep intervention, either digital or face-to-face depending on student preferences, that takes into account their unique circumstances and specific needs in relation to sleep. Through a series of focus groups, the primary goal of this study was to garner an understanding of student views and preferences on program format and content for a sleep-focused intervention. Subsidiary goals were to understand student concerns about sleep and associated help-seeking behavior for sleep.

## Methods

### Design

A series of 11 focus groups were conducted via videoconferencing. Participant numbers in each group were limited to 4, to allow each individual time to share their experiences and facilitate the sharing of personal information [46].

### Participants

Participants were university students at the University of New South Wales, Sydney (New South Wales, Australia), who took part in the study in exchange for course credit (during the semester) or an AUD $25 (US $16.58) gift voucher (during vacation). They were recruited between May and July 2020 through a university-based research participation portal for all undergraduate students. Participants were only permitted to enroll in the study if they were university students aged 18-25 years. There were no criteria for sleep difficulty level because the goal was to develop a program that would be suitable for students with a wide range of sleep difficulties.

### Measures

#### Demographics Questionnaire and Interview Schedule

Basic demographics including age, gender, degree currently enrolled in, year of enrollment, employment, country of birth, and history of mental illness were assessed. A list of key discussion questions was developed (see Multimedia Appendix 1 for a sample of these questions) and used as a guide to prompt participants to share their views and attitudes. Topics that arose spontaneously were pursued and followed up to gain a richer understanding of university student perspectives and experiences with sleep health and sleep difficulties.

#### Insomnia Severity Index

The Insomnia Severity Index (ISI) [47] is a 7-item measure of insomnia symptoms, where higher scores indicate more severe insomnia. A score below 8 indicates no clinically relevant symptoms of insomnia, scores from 8 to 14 suggest subthreshold insomnia, scores from 15 to 21 indicate clinical insomnia of moderate severity, and scores from 22 to 28 indicate the presence of severe clinical insomnia. The ISI has been validated for use in university students [48]. The ISI also has good reliability; a meta-analysis of 33 studies reported a pooled internal consistency of 0.83 [49]. Internal consistency in the current sample was 0.84.

### Ethics Approval

Ethics approval to conduct this research was granted by the University of New South Wales Human Research Ethics Advisory Panel C: Psychology (HREAP3422).

### Procedure

Participants registered interest through the research participation portal, then read the participant information sheet, and provided consent to participate using the QualtricsXM web-based software platform (SAP America Inc) survey platform. They then completed the demographics questionnaire and ISI [47]. All focus groups were facilitated by the first author—an experienced clinical psychologist. Focus groups ran for 45-60 minutes via videoconferencing due to COVID-19 restrictions preventing in-person meetings. Participants were reassured that their confidentiality would be maintained at the start of focus group sessions, and each participant took part in only 1 focus group.

Semistructured questions (see Multimedia Appendix 1) were devised to elicit discussion so that key research questions would be addressed by the focus groups. The goals were to explore (1) student experiences with sleep difficulties and what they would like a sleep program to address; (2) whether they perceived any stigma associated with seeking help for sleep difficulties; (3) student views on the suitability of an app to improve sleep; (4) preferences for program format, pacing, and content; (5) ideas about motivation and engagement; and (6) views on the use of social media to support an intervention. Focus groups were conducted sequentially using an iterative process until the research questions had been answered and further focus groups were not necessary.

### Analysis

Participants’ characteristics were analyzed and reported using descriptive statistics. Thematic analysis was used to analyze the data from focus group discussions [50]. This flexible approach to qualitative analysis allows data to be analyzed across a large data set and yields a summary of key features of the data. The responses from the focus groups’ questions were analyzed together. The semistructured questions used to guide group discussions were not used as data themes. This approach was taken to avoid imposing a predetermined structure on participant responses and keep the analysis open to unanticipated ideas reflected by the data while integrating the original aims of the data analysis [50].

Transcripts and audio recordings were automatically produced by the videoconferencing software (Zoom; Zoom Technologies, Inc). The transcripts were inaccurate and had to be checked and corrected against the original audio recording, which was performed by the first author. Following this, the entire data set was coded and organized into themes independently by 2 authors (MT and EU). Both coders then collaboratively reviewed the themes and individually coded and analyzed the data until the final analysis was deemed to be an accurate reflection of the discussion content.

## Results

### Participant Characteristics

A total sample of 30 university students (77% female, mean age 20.3, SD 1.89, age range 18-24 years) took part in the focus groups. Participants scored an average of 11.86 (SD 5.77) on the ISI putting them in the mild range with subthreshold insomnia. Participant demographics are shown in Table 1. There was an average of 3 participants per group, with a range from one to 4 participants in each group.

**Table 1:** Participant characteristics (N=30).

Variable			Values
**Age (years)**			
	Mean (SD)		20.33 (1.89)
	Range		18-24
**Gender, n (%)**			
	Male		7 (23)
	Female		23 (77)
**Education, n (%)**			
	Postgraduate		1 (3)
	**Undergraduate**		29 (97)
		First year	14 (4)
		Second year	4 (13)
		Third year	6 (20)
		Fourth year	3 (10)
		Fifth year	2 (7)
**Degree, n**			
	Criminology		4
	Data science		4
	Information technology		3
	Medicine		1
	Psychology		13
	Science and arts		3
	Social work		1
	Unspecified bachelor’s degree		1
**Country of birth, n (%)**			
	Born in Australia		22 (73)
	Born Overseas (India, Taiwan, China, Hong Kong, South Korea, and Pakistan)		8 (27)
**Employment, n (%)**			
	Work (casual or part-time)		21 (70)
	Not working		9 (30)
**Mental health, n (%)**			
	**Experienced or been diagnosed with a mental health problem?**		
		Yes	12 (40)
		No	15 (50)
		Not sure	3 (10)
ISI^a^ total score, mean (SD)			11.86 (5.77)
**Insomnia symptoms, n (%)**			
	Severe insomnia		1 (3)
	Moderate insomnia		9 (30)
	Mild insomnia		11 (37)
	No insomnia		9 (30)

^a^ISI: Insomnia Severity Index.

### Thematic Analysis

The thematic analysis resulted in the identification of three main themes: (1) program development, (2) help-seeking, and (3) student sleep characteristics (see Table 2). Subthemes were identified under each of these main themes and are discussed in detail, along with example quotes, in Table 2.

**Table 2:** Quotes identified by thematic analysis.

Theme and subtheme		Subtheme and theme description	Quotes	Potential intervention features to include
**Program development**				
	Program format	Format considerations that arose included the delivery context, mode of delivery, timing of the intervention, duration, and cost.	*it's just like so much easier to have a conversation about something, because when ... you know it’s reading material and like passively taking information it's just so much harder to process and actually, like take in that information. So yeah, video conferencing or face to face...I think that they're the most effective ways to like actually make something useful.* [Participant #15, female, 18 years old]	Brief intervention (4-6 weeks duration) Small groups or individual therapy Weekly hour-long sessions In-person live component Videoconferencing or on site Expert facilitator
	Program content	A preference was identified for content with scientific information individualized to each student’s unique needs. Students were also interested in learning to manage screen time.	*it's kind of hard to get adequate help from online sources,...it's hard to get help specifically for your situation if it's not in person.* [Participant #2, female 20, years old]	Information on the science of sleep Capacity to tailor to the individual needs Strategies to reduce screen time at bedtime when there is a desire for sleep
	Engagement facilitator	Students would feel more motivated to stay in a program if they had an opportunity to track their progress, set goals, and read content between sessions. A program where they felt socially connected to others through live interactions and social media would be appealing to students.	*say people were told to set a goal at the very start of the program ... and then say like halfway through the program they're given their goals and ... you’d look at it and subconsciously sort of think like what is my progress so far.* [Participant #21, female, 19 years old]	Feedback on progress Facilitate social interaction
**Help-seeking**				
	N/A^a^	Students identified when and where they would get help for a sleep problem. They described how they perceived little stigma associated with seeking help for sleep but did report on other barriers to help-seeking including a lack of awareness of the importance of sleep and available and accessible interventions.	*sleep seems more universal ...I guess, there is less stigma around it.* [Participant #6, male, 20 years old]	Recruit participants by raising awareness among students of the need for help with sleep and the availability of psychological treatments
**Students sleep characteristics**				
	Factors disturbing sleep	Healthy sleep was hard to maintain due to the lack of routine inherent to the student lifestyle. Students’ sleep quality was also diminished by the demands of university study (completing assessments) and the challenge of juggling competing priorities that often involve late nights. Mobile phones and technology use were also damaging to sleep.	*as a student like there's never really a stop time....there's not a clear schedule you can't just leave your work at uni.* [Participant #3, female 20 years old] *it's kind of like hard to balance it all and get a good sleep, well because, it seems like the easiest thing you could cut out.* [Participant #20, male, 18 years old]	Acknowledge the lack of routine and demands of student life Give realistic recommendations for sleep regularity and sleep hygiene Encourage monitoring of technology use
	Consequences of poor sleep	Students found poor sleep had adverse cognitive, emotional, and behavioral effects though keeping busy through the day was helpful.	*I kind of get really irritable and kind of just don't talk to anyone.* [Participant #6, male, 20 years old]	Acknowledge the impact of sleep difficulties.

^a^N/A: not available.

### Program Development

#### Format

Student responses indicated a clear preference for a live socially interactive program with an expert facilitator who could answer questions in real time and actively demonstrate how to develop and implement sleep skills. Participants were largely disinterested in self-guided web-based programs, describing them as hard to engage with and easy to ignore

It's just like so much easier to have a conversation about something, because when... you know it’s reading material and like passively taking information it's just so much harder to process and actually, like take in that information.

Participant #15, female, 18 years old

Similarly, students thought existing mobile apps for sleep were not customized to their needs and were only willing to consider these as an adjunct to a therapist-led program. Since students wanted a face-to-face program, we then explored students’ views on videoconferencing. They felt there were significant benefits, including the convenience and accessibility of the format, and the time-saving aspect of not having to travel to and from an on-site program. Students indicated that if the program was run via videoconferencing that it was essential that cameras were on and that the program was run in an interactive way, with ample encouragement and feedback for them to actively participate.

Students were divided over whether the program should involve one-on-one therapy or a group format. Some students were indifferent and happy with either, while others showed a preference for individual therapy, arguing it would make it easier for people to open up. Conversely, others thought groups would be more helpful and supportive. In addition, there was a very strong preference that group sizes should be small. Small groups were seen as more engaging and comfortable, with a size of around five people thought to be optimal.

Students generally preferred a free program. If there was a fee charged, a maximum cost of about AUD $60 (US $40) would be acceptable, with a higher cost precluding their participation. Participants preferred weekly sessions of about 1 hour’s length as opposed to a more intensive workshop style. An overall duration of 4-6 weeks was seen as optimal, with a maximum duration of about 10 weeks being viewed as acceptable.

#### Content

Respondents indicated a strong desire for individualized assistance that is tailored to their unique situation. Automated web-based interventions were interpreted as very generic. Students also wanted to learn scientific information about sleep and sleep health strategies that were based on research. They also thought that having the intervention delivered by a qualified expert would make the program content more credible.

Some students indicated they would like a sleep program to include help with getting off screens. Phone use had an addictive quality for some students who felt their phone use was interfering with getting to sleep at night: “I really struggle to get off my phone... I like can’t stop” [Participant #16, female, 20 years old].

#### Engagement

Participants suggested that having a visible record of their sleep would help them to track their progress and that seeing positive results from their efforts would motivate them to stay engaged. They also suggested that having a way to compare their progress with others would help to keep them motivated. Students thought that between-session reminders to review the program content would help to keep sleep improvement a priority for them between sessions. They thought this information could be provided via email or a social media platform such as Instagram or Facebook. Students indicated that setting clear goals and then having a reminder of their goals given to them mid-program would motivate them to stay engaged with the program.

The potential to develop relationships with other participants was considered appealing. This would enable support and helpful suggestions from peers and help motivate them to keep participating and working to change their habits:

It's more encouraging to know that you're going to be seeing more people there and like you might make friends there, and you know also work on your problems whilst at home so it's like not exclusive to just those sessions, because if you make a friend they’ll be like how did you sleep last night?

Participant #13, female, 19 years old

A social media component was frequently suggested to help participants feel connected to each other and receive content and reminders between sessions that would facilitate their adherence to the program.

### Help-Seeking

#### When and Where to Access Help

Participants indicated that they would seek help if they were having trouble functioning or noticed that their mood, emotional well-being, concentration, and day-to-day activities were adversely affected by poor sleep. Participants commonly indicated that problems would need to reach a severe level to trigger external help-seeking, as they generally preferred to self-manage. Typically, students identified their general practitioner as the first place they would go to seek help. Others nominated a sleep specialist, doctor, or psychologist. Some students said they would go online or seek medication to improve their sleep.

#### Stigma

Participants indicated little perceived stigma around seeking help for sleep concerns. They had no concerns about others knowing they were seeking professional help for sleep. They did, however, perceive more stigma associated with seeking help for mental health difficulties, such as depression or anxiety.

#### Barriers to Help-Seeking

Students reported thinking of sleep problems as a normal part of student life. They noted that this prevented them from recognizing the need to get help with their sleep. Students expressed a stoic attitude, viewing sleep difficulties as part of being a student, and a problem they needed to self-manage. Students also lacked awareness of the longer-term effects of poor sleep and many thought that having a greater knowledge of this would increase their motivation to improve their sleep. Despite the prevalence of sleep problems, participants noted that it was rarely something they heard about, thought about, or discussed with their peers,

Maybe a lot of people wouldn't seek help as well because they just think oh everyone's in the same boat like, that's just the life of a uni student you just have to suck it up, kind of thing, and they might not realise how serious their issues are.

Participant #1, female, 20 years old

Some students blamed themselves for their poor sleep. Although they identified the lack of structure and routine in their lifestyle, they felt they should be able to overcome this with discipline and sheer effort. Students who might consider getting help were concerned that others might not understand the severity of their sleep struggles, while others feared that seeking help would temporarily worsen their sleep, which they felt unable to cope with.

### Student Sleep Characteristics

#### Factors Disturbing Sleep

Students reported that meeting course requirements (eg, study and assignments) frequently interfered with their ability to get a good night’s sleep. Many students described working on assignments late at night when they have improved concentration and productivity. In addition, they described an academic workload that frequently fluctuates, with some intense assessment periods and other phases of extended vacation and no academic demands. At times they felt overwhelmed by their workload and cut out sleep to gain extra time to meet course demands. They also reported prioritizing leisure activities ahead of sleep at times: “You either don’t get sleep or you don’t get to do things you like” (Participant #20, male, 18 years old).

Smartphone and social media use were also acknowledged as contributing to a delay in sleep onset. Many students identified the lack of routine in their lifestyle as a major contributor to poor sleeping patterns. Students described how unstructured university work patterns make switching off from study demands a challenge. They note how they are often able to take naps during the day and work late into the night, and this flexibility enables them to maintain irregular patterns of sleeping and waking, which they find contributes to the maintenance of sleep difficulties.

#### Consequences of Poor Sleep

Students reported that the most typical cognitive effects of poor sleep were a general feeling of daytime sleepiness and poor concentration. Mood effects were common with students typically describing increased irritability. They also felt more withdrawn in social situations and found they were less talkative.

Following poor sleep, students described avoiding effortful tasks, increasing their caffeine consumption, napping more frequently, and being prone to overeating. Some students recognized how these behaviors were contributing to maintaining their sleep difficulties,

I fall asleep on trains and buses and always take random naps in the afternoons and evenings that I don't really want to take, that facilitates a pretty vicious cycle, because then you can't sleep at an earlier hour.

Participant #11, female, 24 years old

Students often noted that the effects of poor sleep were intermittent and did not consistently follow a bad night’s sleep. For example, the impact of poor sleep was more clearly felt on a quiet day than on a busy day.

#### Summary

Taken together, the results from the thematic analysis indicated that students felt an intervention for sleep should be tailored to university students’ needs and have an in-person expert facilitator who can respond to individual circumstances and questions. They also indicated that a way to monitor their progress was important to enhance motivation. It was clear that help-seeking for sleep issues was not stigmatizing. Finally, students identified aspects unique to their student lifestyle (workload, hours, and lack of consistent schedules) as contributors to their sleep issues.

## Discussion

The purpose of this research was to engage in a user-centered approach to develop a new sleep intervention to meet the unique needs of university students. The main findings resulting from a series of focus groups indicated that participants advocated for an intervention with social interaction that was delivered either one on one or in small groups. They were keen to have opportunities for in-person interaction with the facilitator and their peers throughout the program and were most in favor of weekly hour-long sessions of about 4 to 6 weeks duration. They saw web-based material or a mobile phone app as a helpful aide to a digital program and wanted a sleep program that was customized to their unique situation and would give recommendations that were realistic and suitable for the university student lifestyle.

A clear theme was that students found the lack of a consistent routine contributed significantly to difficulties with sleep. The fluctuating demands of university study along with the frequent late nights caused by finishing assignments, working in casual jobs, and socializing, regularly interfered with their sleep patterns. This finding led to the suggestion that an appropriate intervention designed for university students needed to be flexible and provide information about the consequences of shifts in routine rather than a blanket insistence on adherence to regular sleep times. Other issues students indicated an interest in being addressed included managing screen time and getting sufficient sleep during examination periods. To the best of our knowledge, there are currently no evidence-based sleep-focused interventions for young adult university students that specifically take these factors into account [36]. This highlights the need for a program to improve sleep in university students that consider the unique circumstances of the student lifestyle.

A strong preference for a live socially interactive program format was a recurring theme throughout the focus group discussions. This format was considered ideal because it allows for social interaction with the facilitator and peers. A need for social connection was identified as a theme, evidenced by the suggestion for a social media component of the program. They viewed this as an opportunity to develop peer relationships, which they thought would help maintain their motivation and engagement with the program. The centrality of social relationships for young adult university students and the importance of including social components when designing mental health interventions has been identified in previous research exploring the needs of university students in mental health interventions [51].

This overall finding that students preferred a program with real-time communication and interaction with a facilitator is consistent with previous research that shows the majority of adults [52] and university students [53-55] still prefer seeking out face-to-face support compared to receiving treatment on the internet [53] and e–mental health services [52,54]. This is despite the research showing that university students are willing to use digital interventions [56]. There is some evidence that students’ preference for face-to-face interventions could be influenced by the type of mental health concern they are seeking help for [57]. For example, past research has found that where people are seeking help for a problem that they perceive as less stigmatized, there is a preference for face-to-face interventions. In contrast, if they perceive the problem is highly stigmatized, then web-based interventions are preferred [57]. This could be explained by the greater privacy and anonymity that are possible with web-based interventions, which makes them particularly appealing when stigma is a barrier to accessing treatment. In this study, student responses aligned with earlier research [58] that there is less stigma associated with seeking help for sleep difficulties relative to other mental health problems. This may account for preferences for in-person treatment in this study.

Results from this study suggested that digital interventions requiring a self-directed approach were considered to be less engaging and too easy to ignore. Considering how much time students already spend engaging in self-directed learning [59], it is not surprising that interventions also requiring students to independently work through material may be unappealing. Students’ perception that they would more easily disengage with an unguided digital intervention is also consistent with the low levels of adherence commonly found in such interventions [60-62].

There are several study limitations that need to be considered. The participants in this study had a range of sleep difficulties, from no sleep difficulties to severe insomnia. The sample did contain very few (n=1, 3%) participants with severe sleep difficulties. The sample was also largely composed of females (n=23, 77%) studying psychology (n=13, 43%). Therefore, the findings of this study may not accurately represent the views of male students, those studying in other fields, and those with more extreme sleep disturbances. In addition, the focus groups were conducted in 2021 during the COVID-19 pandemic in Sydney during a period of sustained lockdown. Although speculative, the social isolation during this time could have been a factor in the results of this study, particularly the student preferences for a face-to-face interactive program and the theme of students seeking social interaction and opportunities to connect in the context of a sleep intervention.

The results from this study indicate that university students feel that the unique aspects of the student lifestyle contribute to sleep difficulties. In their view, an appropriate intervention should involve interactions in real time, either in person or via videoconferencing, specifically tailored to their lifestyles, and with a social component. These findings can help inform the development of psychological sleep interventions for this high-risk university student population.

## Appendix

Multimedia Appendix 1 Sample discussion questions for focus groups.

## Abbreviations

CBT-I: cognitive behavioral therapy for insomnia
DSM-V: Diagnostic and Statistical Manual of Mental Disorders Fifth Edition
ISI: Insomnia Severity Index
RCT: randomized controlled trial

## References

[1] Lund HG, Reider BD, Whiting AB, Prichard JR (2010). Sleep patterns and predictors of disturbed sleep in a large population of college students. J Adolesc Health.

[2] Becker SP, Jarrett MA, Luebbe AM, Garner AA, Burns GL, Kofler MJ (2018). Sleep in a large, multi-university sample of college students: sleep problem prevalence, sex differences, and mental health correlates. Sleep Health.

[3] (2013). Diagnostic and statistical manual of mental disorders, fifth edition. American Psychiatric Association.

[4] Jiang XI, Zheng X, Yang J, Ye C, Chen Y, Zhang Z, Xiao Z (2015). A systematic review of studies on the prevalence of insomnia in university students. Public Health.

[5] Ohayon MM (2002). Epidemiology of insomnia: what we know and what we still need to learn. Sleep Med Rev.

[6] Hershner S (2020). Sleep and academic performance: measuring the impact of sleep. Curr Opin Behav Sci.

[7] Vedaa Ø, Erevik EK, Hysing M, Hayley AC, Sivertsen B (2019). Insomnia, sleep duration and academic performance: a national survey of Norwegian college and university students. Sleep Med X.

[8] Gaultney JF (2010). The prevalence of sleep disorders in college students: impact on academic performance. J Am Coll Health.

[9] Simon EB, Walker MP (2018). Sleep loss causes social withdrawal and loneliness. Nat Commun.

[10] Bernert RA, Kim JS, Iwata NG, Perlis ML (2015). Sleep disturbances as an evidence-based suicide risk factor. Curr Psychiatry Rep.

[11] Pigeon WR, Bishop TM, Titus CE (2016). The relationship between sleep disturbance, suicidal ideation, suicide attempts, and suicide among adults: a systematic review. Psychiatric Annals.

[12] Pigeon WR, Bishop TM, Krueger KM (2017). Insomnia as a precipitating factor in new onset mental illness: a systematic review of recent findings. Curr Psychiatry Rep.

[13] Lovato N, Gradisar M (2014). A meta-analysis and model of the relationship between sleep and depression in adolescents: recommendations for future research and clinical practice. Sleep Med Rev.

[14] Freeman D, Sheaves B, Goodwin GM, Yu LM, Nickless A, Harrison PJ, Emsley R, Luik AI, Foster RG, Wadekar V, Hinds C, Gumley A, Jones R, Lightman S, Jones S, Bentall R, Kinderman P, Rowse G, Brugha T, Blagrove M, Gregory AM, Fleming L, Walklet E, Glazebrook C, Davies EB, Hollis C, Haddock G, John B, Coulson M, Fowler D, Pugh K, Cape J, Moseley P, Brown G, Hughes C, Obonsawin M, Coker S, Watkins E, Schwannauer M, MacMahon K, Siriwardena AN, Espie CA (2017). The effects of improving sleep on mental health (OASIS): a randomised controlled trial with mediation analysis. Lancet Psychiatry.

[15] Baglioni C, Battagliese G, Feige B, Spiegelhalder K, Nissen C, Voderholzer U, Lombardo C, Riemann D (2011). Insomnia as a predictor of depression: a meta-analytic evaluation of longitudinal epidemiological studies. J Affect Disord.

[16] Liu RT, Steele SJ, Hamilton JL, Do QBP, Furbish K, Burke TA, Martinez AP, Gerlus N (2020). Sleep and suicide: a systematic review and meta-analysis of longitudinal studies. Clin Psychol Rev.

[17] Dombrovski AY, Cyranowski JM, Mulsant BH, Houck PR, Buysse DJ, Andreescu C, Thase ME, Mallinger AG, Frank E (2008). Which symptoms predict recurrence of depression in women treated with maintenance interpersonal psychotherapy?. Depress Anxiety.

[18] Bei B, Asarnow LD, Krystal A, Edinger JD, Buysse DJ, Manber R (2018). Treating insomnia in depression: insomnia related factors predict long-term depression trajectories. J Consult Clin Psychol.

[19] Pigeon WR, Hegel M, Unützer J, Fan MY, Sateia MJ, Lyness JM, Phillips C, Perlis ML (2008). Is insomnia a perpetuating factor for late-life depression in the IMPACT cohort?. Sleep.

[20] Buysse DJ, Tu XM, Cherry CR, Begley AE, Kowalski J, Kupfer DJ, Frank E (1999). Pretreatment REM sleep and subjective sleep quality distinguish depressed psychotherapy remitters and nonremitters. Biol Psychiatry.

[21] Espie CA, Morin CM, Morin CM, Espie CA (2012). Introduction: historical landmarks and current status of sleep research and practice: an introduction to the timelines, aims and scope of this handbook. The Oxford Handbook of Sleep and Sleep Disorders.

[22] Ramar K, Malhotra RK, Carden KA, Martin JL, Abbasi-Feinberg F, Aurora RN, Kapur VK, Olson EJ, Rosen CL, Rowley JA, Shelgikar AV, Trotti LM (2021). Sleep is essential to health: an American academy of sleep medicine position statement. J Clin Sleep Med.

[23] McGorry PD, Sartorius N, de Girolama G, de Girolama G, McGorry PD, Sartorius N (2019). Conclusions: from the study of the age of onset to the development of age-specific interventions in mental health. Age of Onset of Mental Disorders: Etiopathogenetic and Treatment Implications.

[24] Patel V, Flisher AJ, Hetrick S, McGorry P (2007). Mental health of young people: a global public-health challenge. Lancet.

[25] Stallman HM (2010). Psychological distress in university students: a comparison with general population data. Aust Psychol.

[26] Dzierzewski JM, Griffin SC, Ravyts S, Rybarczyk B (2018). Psychological interventions for late-life insomnia: current and emerging science. Curr Sleep Med Rep.

[27] Haynes J, Talbert M, Fox S, Close E (2018). Cognitive behavioral therapy in the treatment of insomnia. South Med J.

[28] Murawski B, Wade L, Plotnikoff RC, Lubans DR, Duncan MJ (2018). A systematic review and meta-analysis of cognitive and behavioral interventions to improve sleep health in adults without sleep disorders. Sleep Med Rev.

[29] Trauer JM, Qian MY, Doyle JS, Rajaratnam SMW, Cunnington D (2015). Cognitive behavioral therapy for chronic insomnia: a systematic review and meta-analysis. Ann Intern Med.

[30] Mitchell MD, Gehrman P, Perlis M, Umscheid CA (2012). Comparative effectiveness of cognitive behavioral therapy for insomnia: a systematic review. BMC Fam Pract.

[31] Ree M, Junge M, Cunnington D (2017). Australasian sleep association position statement regarding the use of psychological/behavioral treatments in the management of insomnia in adults. Sleep Med.

[32] Schutte-Rodin S, Broch L, Buysse D, Dorsey C, Sateia M (2008). Clinical guideline for the evaluation and management of chronic insomnia in adults. J Clin Sleep Med.

[33] van Straten A, van der Zweerde T, Kleiboer A, Cuijpers P, Morin CM, Lancee J (2018). Cognitive and behavioral therapies in the treatment of insomnia: a meta-analysis. Sleep Med Rev.

[34] Perlis ML, Posner D, Riemann D, Bastien CH, Teel J, Thase M (2022). Sleep and sleep disorders 2: insomnia. Lancet.

[35] Friedrich A, Schlarb AA (2018). Let's talk about sleep: a systematic review of psychological interventions to improve sleep in college students. J Sleep Res.

[36] Saruhanjan K, Zarski AC, Bauer T, Baumeister H, Cuijpers P, Spiegelhalder K, Auerbach RP, Kessler RC, Bruffaerts R, Karyotaki E, Berking M, Ebert DD (2021). Psychological interventions to improve sleep in college students: a meta-analysis of randomized controlled trials. J Sleep Res.

[37] Fucito LM, DeMartini KS, Hanrahan TH, Yaggi HK, Heffern C, Redeker NS (2017). Using sleep interventions to engage and treat heavy-drinking college students: a randomized pilot study. Alcohol Clin Exp Res.

[38] Morris J, Firkins A, Millings A, Mohr C, Redford P, Rowe A (2016). Internet-delivered cognitive behavior therapy for anxiety and insomnia in a higher education context. Anxiety Stress Coping.

[39] Miller MB, Deroche CB, Freeman LK, Park CJ, Hall NA, Sahota PK, McCrae CS (2021). Cognitive behavioral therapy for insomnia among young adults who are actively drinking: a randomized pilot trial. Sleep.

[40] Taylor DJ, Zimmerman MR, Gardner CE, Williams JM, Grieser EA, Tatum JI, Bramoweth AD, Francetich JM, Ruggero C (2014). A pilot randomized controlled trial of the effects of cognitive-behavioral therapy for insomnia on sleep and daytime functioning in college students. Behav Ther.

[41] Foulkes L, McMillan D, Gregory AM (2019). A bad night's sleep on campus: an interview study of first-year university students with poor sleep quality. Sleep Health.

[42] Gardani M, Bradford DRR, Russell K, Allan S, Beattie L, Ellis JG, Akram U (2022). A systematic review and meta-analysis of poor sleep, insomnia symptoms and stress in undergraduate students. Sleep Med Rev.

[43] Wang F, Bíró É (2021). Determinants of sleep quality in college students: a literature review. Explore (NY).

[44] Farao J, Malila B, Conrad N, Mutsvangwa T, Rangaka MX, Douglas TS (2020). A user-centred design framework for mHealth. PLoS One.

[45] Lyon AR, Koerner K (2016). User-centered design for psychosocial intervention development and implementation. Clin Psychol (New York).

[46] Morgan DL, Hoffman K, Flick U, Focus Groups (2018). The SAGE Handbook of Qualitative Data Collection.

[47] Morin CM, Belleville G, Bélanger L, Ivers H (2011). The insomnia severity index: psychometric indicators to detect insomnia cases and evaluate treatment response. Sleep.

[48] Wong ML, Lau KNT, Espie CA, Luik AI, Kyle SD, Lau EYY (2017). Psychometric properties of the sleep condition indicator and insomnia severity index in the evaluation of insomnia disorder. Sleep Med.

[49] Cerri LQ, Justo MC, Clemente V, Gomes AA, Pereira AS, Marques DR (2023). Insomnia severity index: a reliability generalisation meta-analysis. J Sleep Res.

[50] Braun V, Clarke V (2019). Reflecting on reflexive thematic analysis. Qual Res Sport Exerc Health.

[51] Lattie EG, Kornfield R, Ringland KE, Zhang R, Winquist N, Reddy M (2020). Designing mental health technologies that support the social ecosystem of college students. https://europepmc.org/abstract/MED/32656549.

[52] March S, Day J, Ritchie G, Rowe A, Gough J, Hall T, Yuen CYJ, Donovan CL, Ireland M (2018). Attitudes toward e-mental health services in a community sample of adults: online survey. J Med Internet Res.

[53] Horgan A, Sweeney J (2010). Young students' use of the internet for mental health information and support. J Psychiatr Ment Health Nurs.

[54] Klein B, Cook S (2010). Preferences for e-mental health services amongst an online Australian sample. E-J Appl Psychol.

[55] Karwig G, Chambers D (2016). E-mental health on campus: college students' views of online help seeking. Annu Rev Cybertherapy Telemed.

[56] Montagni I, Tzourio C, Cousin T, Sagara JA, Bada-Alonzi J, Horgan A (2020). Mental health-related digital use by university students: a systematic review. Telemed J E Health.

[57] Wallin E, Maathz P, Parling T, Hursti T (2018). Self-stigma and the intention to seek psychological help online compared to face-to-face. J Clin Psychol.

[58] Ribeiro JD, Pease JL, Gutierrez PM, Silva C, Bernert RA, Rudd MD, Joiner TE (2012). Sleep problems outperform depression and hopelessness as cross-sectional and longitudinal predictors of suicidal ideation and behavior in young adults in the military. J Affect Disord.

[59] Sumuer E (2018). Factors related to college students’ self-directed learning with technology. Australas J Educ Technol.

[60] Christensen H, Griffiths KM, Farrer L (2009). Adherence in internet interventions for anxiety and depression. J Med Internet Res.

[61] Donkin L, Christensen H, Naismith SL, Neal B, Hickie IB, Glozier N (2011). A systematic review of the impact of adherence on the effectiveness of e-therapies. J Med Internet Res.

[62] Horsch C, Lancee J, Beun RJ, Neerincx MA, Brinkman WP (2015). Adherence to technology-mediated insomnia treatment: a meta-analysis, interviews, and focus groups. J Med Internet Res.

