# Supplementary material for: Preferences of University Students for a Psychological Intervention Designed to Improve Sleep: Focus Group Study
Source: JMIR Hum Factors. 2023 Aug 24;10:e44145. doi: 10.2196/44145 (PMC10485721; doi:10.2196/44145)
Supplement: Multimedia Appendix 1 [file humanfactors_v10i1e44145_app1.docx]

**1. General questions**

- Tell me about your sleep?

optional prompts:

Have you gone through periods where you have not slept well?

Are there things in your environment that make it difficult to sleep?

Do thoughts and worries sometimes make it hard to sleep?

Is your sleep interrupted by social activities, study, casual work?

Routine?

- How are you effected by not sleeping well? [optional prompts: when you are not sleeping well: is your concentration effected? Are your social interactions different? Is your mood different?
- How do you feel about not sleeping well? [optional prompt: Is it something that worries you?]
- At what point would you get help for sleep?
- Where would you go for help ?
- Do you have any concerns about seeking help for sleep problems?
- How would you feel about other people knowing you are taking part in a program to improve your sleep?
- Do you have any concerns about seeking help for emotional problems?
- How would you feel about other people knowing you are taking part in a program to improve your mental health?
- What would be good about improving sleep?

**2. Program Content**

- What are some areas of your life that are particularly important to you (for eg do you care most about your physical health, friendships, studies, the future, emotional wellbeing, recreation, sport, religion, finances) ?
- Tell us more about why those areas of your life are important to you?
- Are there aspects of sleep you would like to know more about?
- Are you interested in improving your sleep? If so what are some of the reasons you would like to improve your sleep?
- What would increase your motivation to improve your sleep?
- Would you be willing to take part in sleep restriction?

**3. Program format**

- If you were taking part in a program to help improve your sleep, what form would you like the program to take? [ prompt if needed e.g. individual one on one sessions with a psychologist, group face to face sessions, online learning, mobile phone App?]
- If the program was online, would you like to have contact with a support person? [prompts: what kind of contact would you prefer email, face to face, phone, text message? How often would you like this contact?]
- For face to face programs would you like spaced out weekly sessions or an intensive program delivered in a short period of time, like a workshop over a couple of days?
- Would you be interested in a combined f2f and phone App? ( would you prefer most video conferencing, individual group live f2f
- How long do you think you would stick with it? Why/why not?
- If you were designing a program for your peers, how would you go about keeping people interested?
- How much time would you be willing to spend on a program to improve your sleep?
- Would you be interested in an App for sleep problems?
